# Phytochemical composition and antimicrobial activity of *Matricaria chamomilla* ethanolic extracts against clinical bacterial isolates in Ibb City, Yemen

**DOI:** 10.1038/s41598-026-38001-z

**Published:** 2026-02-03

**Authors:** Abeer Esmail, Asmaa Hassan, Khadeja Almuntaser, Nasibh Alashari, Nosiubah Alkadry, Reem Alnahi, Halah Alhagwa, Rehab Hamza, Nadia Alhubaishy, Ammar AL-Farga, Tasneem Alnuzaili

**Affiliations:** 1 Medical Laboratories Department, Faculty of Medicine and Health Sciences, Ibb University, Ibb City, Yemen; 2 Department of Medical Laboratories, Faculty of Medical Sciences, Al-Jazeera University, Ibb City, Yemen; 3 Department of Pharmacy, Faculty of Medical Sciences, Al-Jazeera University, Ibb City, Yemen; 4 Department of Biochemistry, College of Science, University of Jeddah, Jeddah, Saudi Arabia

**Keywords:** Matricaria chamomilla, Antimicrobial activity, (MIC), (MBC), Phytochemicals, Chamomile, Yemen, Biochemistry, Drug discovery, Microbiology, Plant sciences

## Abstract

This study aimed to analyze the phytochemical constituents and evaluate the antimicrobial effects of ethanolic extracts from *Matricaria chamomilla* (chamomile) leaves and flowers against clinical bacterial isolates collected from Ibb City, Yemen. Phytochemical screening revealed presence of alkaloids, tannins, sterols, and terpenoids in both leaves and flowers, while flavonoids, glycosides, saponins, and amino acids were absent. Antibacterial activity was assessed using inhibition zone diameters against pathogens including *Staphylococcus aureus*, *Escherichia coli*, *Salmonella* spp., and *Pseudomonas aeruginosa*. The extracts showed significant antimicrobial activity, especially against *S. aureus*, with leaf extract showing a 24 mm inhibition zone and flower extract 15 mm. Minimum inhibitory concentration (MIC) and minimum bactericidal concentration (MBC) tests confirmed the potent bactericidal effects at low extract concentrations (100 mg/mL). These findings underscore the therapeutic potential of *M. chamomilla* as a natural antimicrobial agent relevant to combating bacterial infections, especially in the local Yemeni context. Further studies are recommended to isolate active compounds and evaluate mechanisms of action.

## Introduction

Bacterial infections remain a major public health concern worldwide, exacerbated by rising antibiotic resistance that threatens effective treatment of infectious diseases (World Health Organization^1^. This growing concern has prompted a global search for novel therapeutic agents, particularly those derived from natural sources. Medicinal plants, which have been used for centuries in traditional healing systems, are now being re-examined through scientific lenses for their antimicrobial properties^2^.

A variety of medicinal and non-medicinal plant extracts have proven promising antimicrobial activity. For example, the essential oil of *Foeniculum vulgare* (fennel) showed significant inhibitory effects against pathogenic bacterial strains, highlighting its potential as a natural antibacterial agent. Essential oils from *Rosmarinus officinalis* (rosemary) and *Lavandula officinalis* (lavender) demonstrated substantial bacteriostatic activity, particularly against multidrug-resistant bacteria^3,4^. Agro-industrial byproducts such as olive mill wastewater (OMWW) from Fez-Boulman have also shown antimicrobial potential by effectively inhibiting several pathogenic bacteria^5^.

Likewise, essential oil of *Origanum vulgare* (oregano), extracted from the Ouazzane region of Morocco, showed broad-spectrum antimicrobial efficacy against resistant clinical isolates^6^. Furthermore, recent in vitro studies on *Jatropha variegata* (Euphorbiaceae) demonstrated strong antimicrobial effects against clinical isolates from Yemen, positioning this plant as a promising candidate for natural antimicrobial development^7^.

*Matricaria chamomilla* L. (German Chamomile), a well-known member of the Asteraceae family, is widely used in traditional medicine across Europe, Asia, and the Middle East. Its therapeutic applications range from treating gastrointestinal disturbances and skin inflammation to promoting relaxation and sleep. Chamomile is rich in pharmacologically active compounds such as flavonoids, terpenoids (notably α-bisabolol and chamazulene), and essential oils, which contribute to its diverse biological effects including antioxidant, anti-inflammatory, and antimicrobial activities^8–11^.

Several studies have highlighted the antimicrobial efficacy of *M. chamomilla* extracts and essential oils against a variety of Gram-positive and Gram-negative bacteria as well as fungi^10,11^. However, the extent of antimicrobial activity can vary significantly depending on factors such as the plant part used, geographic origin, extraction method, and concentration. Moreover, few studies have focused on comparing the antimicrobial potential of various parts of the chamomile plant against clinically relevant pathogens, especially in regions like Yemen where traditional medicine plays a significant role in healthcare.

Therefore, the current study was designed to assess the in vitro antibacterial activities of ethanolic extracts from the leaves and flowers of *M. chamomilla* collected from Ibb governorate, Yemen, against clinical isolates of *Staphylococcus aureus*, *Escherichia coli*, *Salmonella* spp., and *Pseudomonas aeruginosa*. Additionally, this study aimed to evaluate the phytochemical composition of the extracts and determine their minimum inhibitory concentrations (MIC) and minimum bactericidal concentrations (MBC).

## Materials and methods

### Plant collection and authentication

Leaves and flowers of *Matricaria chamomilla* were collected from the Assabal region, Ibb Governorate, Yemen, on 7 November 2022. The taxonomic identity of the plant was verified by Dr. Essam Aqlan (ORCID: 0000-003-3081-6361), Assistant Professor of Plant Taxonomy and Flora, Department of Biology, Faculty of Science, Ibb University. A voucher specimen (code JV202210/2) was deposited in the Herbarium of the Faculty of Medical Sciences, Al-Jazeera University.

After collecting, the plant materials were carefully washed, air-dried in the shade at room temperature for about four weeks, and then ground into a fine powder for extraction. The procedures for cleaning, shade-drying, and powdering the plant materials prior to extraction followed the methods described in our previous study.

With minor modifications^7^. The plant material used in this study was collected and used following all relevant institutional, national, and international guidelines and legislation.

### Preparation of ethanolic extracts

Ethanolic extraction was performed following the protocol reported in our previous study^12^, with adjustments. Briefly, 95 g of powdered leaves and 95 g of powdered flowers were separately immersed in 450 mL of 96% ethanol and shaken intermittently for 72 h. After filtration, the plant residues were re-macerated under the same conditions. The combined filtrates were evaporated at 40 °C in a hot-air oven, and the dried extracts were preserved in amber bottles at room temperature until use.

### Phytochemical screening

Qualitative phytochemical screening was conducted using standard protocols^13^, adapted from our earlier work^7^. The extracts were examined for the presence of secondary metabolites including alkaloids, glycosides, phenolics, flavonoids, tannins, terpenoids, saponins, carbohydrates, and proteins^14–16^.

### Clinical bacterial isolates

Clinical isolates were obtained from routine diagnostic laboratories and subsequently re-identified using conventional microbiological procedures, as outlined previously^7^. The identified strains included *Staphylococcus aureus*,* Escherichia coli*,* Salmonella* spp., and *Pseudomonas aeruginosa*. Confirmation was achieved through Gram staining, microscopic examination, and biochemical assays in accordance with Bergey’s Manual of Determinative Bacteriology^17^.

### Antimicrobial activity assay

The antimicrobial potential of the ethanolic extracts was evaluated using the agar well diffusion technique, adapted from our earlier study^7^.

- Culture media and Inocula:

Mueller–Hinton agar (MHA) was used for bacterial cultures. Bacterial suspensions were standardized to the 0.5 McFarland turbidity standard (~ 1.5 × 10⁸ CFU/mL).

- Extract solutions:

Stock solutions were prepared by dissolving 0.5 g of each crude extract in 1 mL of 10% dimethyl sulfoxide (DMSO).

- Well diffusion assay:

Wells of 6 mm were created in MHA plates and filled with 50 µL of extract solution. Plates were incubated at 37 °C for 18–24 h, and inhibition zones were measured in millimeters.

### Minimum inhibitory concentration (MIC) and minimum bactericidal concentration (MBC)

MIC and MBC were determined according to Clinical and Laboratory Standards Institute (CLSI) guidelines^18^.

- MIC determination:

Serial dilutions (500–100 mg/mL) of the extracts were prepared in 10% DMSO. Each dilution (1.5 mL) was mixed with 13.5 mL of molten MHA at 55 °C, poured into Petri dishes, and inoculated with 2 µL of bacterial suspension (~ 2 × 10^5^ CFU/spot). Plates having only DMSO served as negative controls. MIC was defined as the lowest extract concentration that completely inhibited visible growth after incubation at 37 °C for 24 h.

- MBC determination:

Aliquots from MIC plates showing no visible growth were streaked onto fresh MHA and incubated for 24 h at 37 °C. The lowest concentration that yielded no bacterial colonies was taken as the MBC, representing ≥ 99.9% killing of the initial inoculum.

## Results

### Extract yield

The extract yield percentage was calculated using the following formula:

$${\text{Percentage Yield }}=\frac{{{\mathrm{Weight~of~Dried~Extract}}}}{{{\mathrm{~~Weight~of~Dried~Plant~Sample~}}}}{\mathrm{~}} \times {\mathrm{~}}100.$$

Based on this calculation, the ethanolic extracts of *Matricaria chamomilla* yielded 12.6% from leaves and 7.85% from flowers (Table 1). These yields reflect the efficiency of ethanol in extracting bioactive compounds from various parts of the plant.

**Table 1:** Yield percentage of *M. chamomilla* extracts.

Extract type	Yield (%)
*M. chamomilla* leaves	12.6
*M. chamomilla* flowers	7.85

### Phytochemical composition

Phytochemical screening showed that both the leaves and flowers had important levels of tannins, alkaloids, sterols, and terpenoids. No flavonoids, glycosides, saponins, or amino acids were detected in either extract (Table 2).

**Table 2:** Phytochemical components are detected in *M. chamomilla* ethanol extracts.

Phytochemical Component	Leaves	Flowers
Alkaloids	++	++
Tannins	++	++
Sterols	++	++
Terpenoids	++	++
Flavonoids	-	–
Glycosides	-	–
Saponins	-	–
Amino acids	-	–

Legend: (++) Present; (–) Absent.

### Antimicrobial activity

The ethanolic extracts of *Matricaria chamomilla* displayed selective antimicrobial activity against the tested microorganisms (Table 3). The leaf extract showed the highest inhibition zone against *Staphylococcus aureus* (24 mm), significantly exceeding that of the flower extract (15 mm), with a mean value of 19.5 mm (SD = ± 6.36 mm). In contrast, the flower extract was more active against *Escherichia coli* (17 mm), while the leaf extract showed no measurable inhibition observed under the tested conditions (0 mm), resulting in the widest variation among the tested organisms (mean = 8.5 mm; SD = ± 12.02 mm). Both extracts produced equal inhibition zones against *Salmonella* spp. (15 mm), showing no variation (SD = 0). For *Pseudomonas aeruginosa*, the leaf extract demonstrated moderate activity (15 mm), while the flower extract was inactive, yielding a mean of 7.5 mm (SD = ± 10.61 mm).

**Fig. 1:**
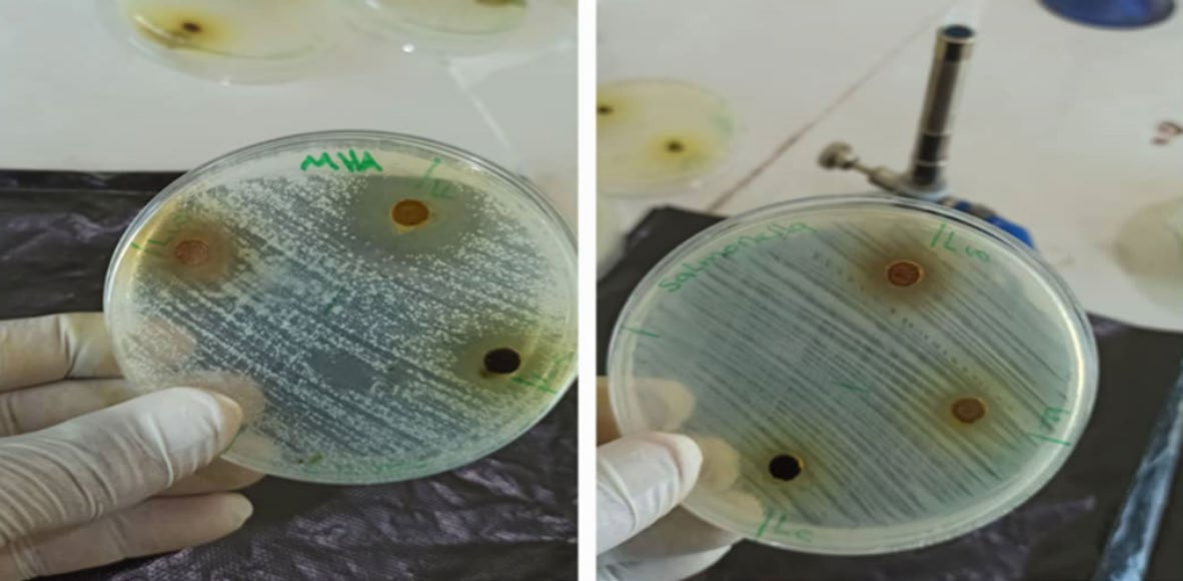
Antimicrobial activity of ethanolic extracts of *Matricaria chamomilla* leaves and flowers against clinical bacterial isolates.

Overall, the results show that the antimicrobial activity of *M. chamomilla* extracts is both organism- and extract-type dependent, with statistically notable differences in mean inhibition zones and standard deviations, especially for *E. coli* and *P. aeruginosa*. (Fig. 1; Table 3)

**Table 3:** Zones of Inhibition (mm), Mean ± SD for **Antibiotic** *and M. chamomilla* extracts.

Antimicrobials/ microbes	Antibiotic IZ (mm)										Extracts IZ (mm)		Control
	Azm 15	Gen 10	CIP 5	OF 5	COT 75	CFM 5	LE 5	NX 10	IPM 10	Mean ± SD	LE Mean ± SD	FL Mean ± SD	DMSO IZ (mm)
*S. aureus*	28	24	24	26	24	11	28	22	26	23.7 ± 10.8	24 ± 1	15 ± 1	0
*E. coli*	24	24	24	40	24	R	20	34	24	23.8 ± 4.5	0 ± 0	17 ± 1	0
*Salmonella spp*	15	25	25	30	26	25	24	30	28	25.3 ± 6.4	15 ± 1	15 ± 1	0
*P. aeruginosa*	12	12	R	R	R	R	R	R	14	4.2 ± 5.1	15 ± 0	0 ± 0	0

Where: IZ: inhibition zone, Azm15: azithromycin, Gen: gentamicin, CIP5: ciprofloxacin, OF5: ofloxacin, COT75: clotrimazole, CFM5: cefexime, LE5: levofloxacin, NX10: nalidixic acid, IPM10: impepem L: leaves, F: Flower. Inhibition zones were measured in mm. Values represent mean ± SD of triplicate experiments. R = resistant. IZ = inhibition zone (mm); L = leaves; F = flowers; Values represent mean ± SD of triplicate measurements; 10% DMSO used as solvent control, no inhibition observed; R = resistant.

### Comparison with antibiotics

The ethanolic extracts of *Matricaria chamomilla* displayed selective antimicrobial activity against the tested microorganisms (Table 3). The leaf extract showed the highest inhibition zone against *Staphylococcus aureus* (24 mm), significantly greater than the flower extract (15 mm), with a mean inhibition zone of 19.5 mm (SD = ± 6.36 mm). In contrast, the flower extract was more active against *Escherichia coli* (17 mm), while the leaf extract showed **no measurable inhibition observed under the tested conditions** (0 mm), yielding the highest variability among tested organisms (mean = 8.5 mm; SD = ± 12.02 mm). Both extracts produced equal inhibition zones against *Salmonella* spp. (15 mm) with no variation (SD = 0). For *Pseudomonas aeruginosa*, the leaf extract demonstrated moderate activity (15 mm), while the flower extract was inactive, resulting in a mean of 7.5 mm (SD = ± 10.61 mm). These results show that antimicrobial activity is both organism- and extract-type dependent, with statistically notable differences in inhibition profiles, particularly for *E. coli* and *P. aeruginosa*.

**Fig. 2:**
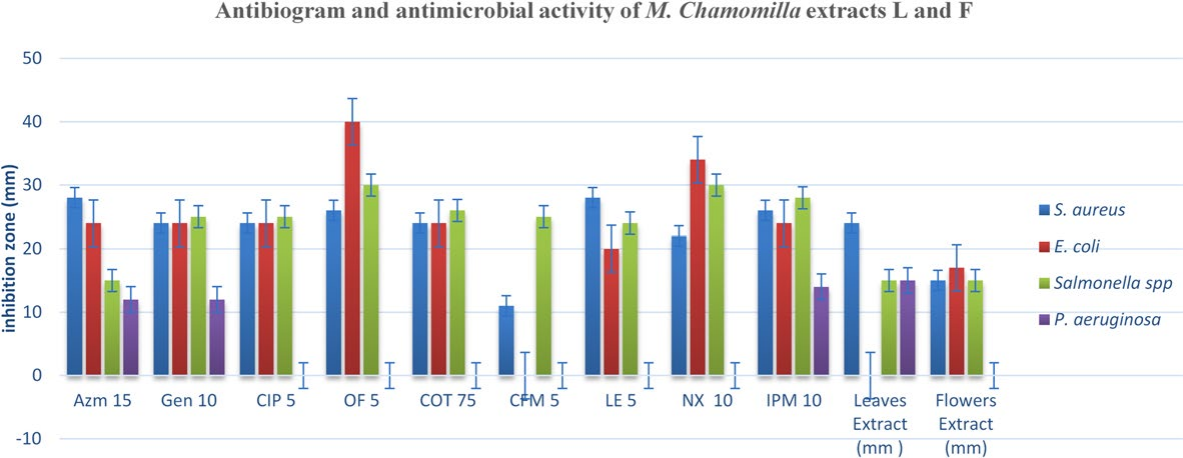
Antibiogram and antimicrobial activity of *M. Chamomilla* extracts leaves and flowers.

When compared with standard antibiotics, *M. chamomilla* extracts showed lower antimicrobial activity, though with some notable exceptions (Table 3). Against *S. aureus*, the leaf extract (24 mm) was comparable to Gentamicin, Amoxicillin, and Cotrimoxazole (24 mm each), and close to Levofloxacin (28 mm) and Azithromycin (28 mm). The flower extract (15 mm) was markedly less effective than all antibiotics. For *E. coli*, the flower extract (17 mm) showed moderate inhibition but remained well below the activity of all antibiotics evaluated, particularly Ciprofloxacin (40 mm), while the leaf extract showed no inhibition. Both extracts inhibited *Salmonella* spp. equally (15 mm), but this was inferior to all antibiotics (≥ 24 mm). Notably, for *P. aeruginosa*, the leaf extract (15 mm) kept moderate activity, surpassing several antibiotics where resistance was observed, and approaching the effect of Imipenem (14 mm), while the flower extract was inactive. These findings suggest that although *M. chamomilla* extracts do not match the potency of conventional antibiotics, the leaf extract demonstrates promising activity against *S. aureus* and *P. aeruginosa*, including strains showing antibiotic resistance. (Table 3; Fig. 2).

### Determination of minimum inhibitory concentration (MIC) and minimum bactericidal concentration (MBC) of plant extract

As shown in Table 4, the *M. chamomilla* flower ethanolic extract showed the same MIC (100 mg/mL) against *Staphylococcus aureus*, *Escherichia coli*, and *Salmonella* spp. This shows that a concentration of 100 mg/mL was sufficient to inhibit the visible growth of all tested bacteria. The MBC values were also 100 mg/mL for all three bacteria, suggesting that the concentration required to kill the organisms was identical to the concentration needed to inhibit their growth. This 1:1 MIC/MBC ratio indicates strong bactericidal activity at the MIC level.

The MIC for the leaf ethanolic extract was also 100 mg/mL for *S. aureus*, *P. aeruginosa*, *E. coli*, and *Salmonella* spp. (Table 5). However, the MBC values differed for some organisms: *P. aeruginosa* required a higher concentration (200 mg/mL) to achieve a bactericidal effect, suggesting a bacteriostatic effect at 100 mg/mL and a bactericidal effect at 200 mg/mL. *S. aureus*,* E. coli*, and *Salmonella* spp. had an MBC equal to the MIC (100 mg/mL), showing bactericidal activity at the MIC concentration.

**Table 4:** Minimum inhibitory concentration and minimum bactericidal concentration of *M. chamomilla* flower extract.

M. chamomilla flowers	MIC mg/mL					MBC mg/mL				
	500	400	300	200	100	500	400	300	200	100
*Staphylococcus aureus*	-	-	-	-	-	-	-	-	-	-
*Salmonella spp*	-	-	-	-	-	-	-	-	-	-
*Escherichia coli*	-	-	-	-	-	-	-	-	-	-

MIC = Minimum inhibitory concentration. (-) no growth, MBC = Minimum bactericidal concentration (-) no growth.

**Table 5:** Minimum inhibitory concentration and minimum bactericidal concentration of *M. chamomilla* leaves extract.

M. chamomilla leaves	MIC mg/mL					MBC mg/mL				
	500	400	300	200	100	500	400	300	200	100
*Staphylococcus aureus*	-	-	-	-	-	-	-	-	-	-
*Pseudomonas aeruginosa*	-	-	-	-	-	-	-	-	-	+
*Salmonella spp*	-	-	-	-	-	-	-	-	-	-

MIC = Minimum inhibitory concentration (-) no growth. MBC = Minimum bactericidal concentration (-) no growth.

The results show that both flower and leaf extracts of *Matricaria chamomilla* exhibit significant antibacterial activity at low concentrations (100 mg/mL), effectively inhibiting the growth of a wide spectrum of bacterial species. The similarity of MIC and MBC values for most bacterial isolates suggests a strong bactericidal effect, especially against *Staphylococcus aureus*, *Escherichia coli*, and *Salmonella* spp. However, the higher MBC observed for *Pseudomonas aeruginosa* when treated with the leaf extract shows a slightly reduced bactericidal efficiency, which may be attributed to its inherent resistance mechanisms or unique cell envelope structure. MIC and MBC determinations were performed in triplicate, and identical results were obtained in all experiments.

## Discussion

*Matricaria chamomilla* (chamomile) is a well-recognized medicinal plant belonging to the Asteraceae family, with over 120 chemical constituents contributing to its diverse biological activities. Numerous studies have demonstrated its antibacterial activity against both Gram-positive and Gram-negative bacteria, primarily attributed to bioactive compounds such as α-bisabolol, luteolin, quercetin, and apigenin, which also provide anti-inflammatory, antiviral, and antioxidant effects^19^. Importantly, this study provides the first MIC and MBC data for *M. chamomilla* collected in Yemen and evaluated against local clinical isolates from Ibb City. While MICs were higher than those reported by Alkuraishy et al. (2015) (2–8 mg/mL) and Mekonnen et al. (2016) (4–16 mg/mL) using standard strains or essential oils, the use of clinically relevant, potentially multidrug-resistant isolates explains the observed differences, highlighting the real-world applicability of our findings^20,21^. This approach strengthens the translational relevance of the findings and better reflects real-world antimicrobial resistance patterns in the local healthcare setting.

Previous research reported antibacterial effects of *M. chamomilla* extracts against Gram-negative strains including *Escherichia coli*, *Pseudomonas aeruginosa*, *Klebsiella pneumoniae*, and *Enterobacter aerogenes*, as well as Gram-positive strains like *Staphylococcus aureus* and *Enterococcus faecalis*, encompassing both methicillin-resistant and methicillin-susceptible isolates. However, the MICs in these studies were relatively high (> 1 mg/mL), indicating limited potency under those experimental conditions^11^.

In the present study, the extraction yields of *M. chamomilla* leaves and flowers were 12.6% and 7.85%, respectively, differing from previous reports such as 20.85% via 50% ethanol maceration and 4.60% via Soxhlet extraction of unprocessed flowers^22^, These variations likely reflect differences in geographic origin, environmental conditions, and extraction methodologies, which also influence the concentration and composition of bioactive phytochemicals and, consequently, antibacterial activity. Importantly, higher yields do not necessarily correlate with greater potency if key antimicrobial constituents are present in low proportions or altered by extraction conditions.

Phytochemical screening revealed elevated levels of tannins, sterols, terpenoids, and alkaloids in both leaves and flowers, whereas saponins, amino acids, flavonoids, and glycosides were absent. These findings partially align with methanolic extract analysis^23^, and chromatographic studies^24^ highlighting the impact of extraction method and plant organ on phytochemical composition. The bioactive compounds exert antibacterial effects through multiple mechanisms, including disruption of the bacterial cell structure leading to increased membrane permeability and leakage of intracellular contents, modification of the cell wall and membrane integrity, depletion of cellular ATP, inhibition of protein synthesis, damage to intracellular components, disturbances in intracellular pH, DNA fragmentation, and interference with bacterial quorum sensing^25–28^.

The absence of flavonoids likely represents the predominant factor underlying the weak susceptibility of *P. aeruginosa* to the extracts, as these compounds constitute the primary bioactive agents exhibiting potent antibacterial activity against this pathogen.

The ethanolic leaf extract demonstrated the highest activity against *S. aureus*, with moderate activity against *E. coli* and *Salmonella* species, showing inhibition zones of 27 mm and 18 mm, respectively, at 400 µg/mL^11^.MIC and MBC values for the flower extract were 100 mg/mL against *S. aureus*, *E. coli*, and *Salmonella*, consistent with other studies reporting MICs of 12.5–50 mg/mL depending on strain and assay method^29^.

Beyond antimicrobial effects, *M. chamomilla* has demonstrated wound-healing potential, improving linear incisional and burn wound repair in experimental animal models^30,31^. This supports the translational relevance of its bioactive compounds for both antimicrobial and regenerative applications. Notably, certain pathogens, including *P. aeruginosa* and *E. faecalis*, were resistant, suggesting strain-specific susceptibility influenced by the absence of flavonoids, low outer membrane permeability, and efflux systems^32^.

Comparative evaluation of leaf versus flower extracts served as an internal control. The higher antibacterial activity of leaves may be attributed to organ-specific differences in the abundance of bioactive constituents, consistent with previous observations in chamomile and other medicinal plants.

In summary, the antibacterial activity observed in this study is mechanistically linked to the phytochemical composition of the extracts, with terpenoids, tannins, and α-bisabolol playing key roles. The organ-specific variation between leaves and flowers, coupled with differences in resistance profiles among bacterial strains, underscores the importance of considering plant part selection and pathogen susceptibility in future investigations.

### Limitation of the study

This study evaluated a limited number of pathogenic strains; thus, findings may not be generalizable across all clinically relevant microbes. Furthermore, in vitro antimicrobial activity does not necessarily translate directly to in vivo efficacy due to complex pharmacokinetic and pharmacodynamic factors influencing bioavailability and interaction with host tissues.

## Conclusions

This study demonstrates that *Matricaria chamomilla* leaves and flowers contain significant bioactive compounds, including tannins, sterols, terpenoids, and alkaloids, which contribute to notable antibacterial activity, particularly against *Staphylococcus aureus*. Importantly, *Pseudomonas aeruginosa*, a multidrug-resistant strain, exhibited sensitivity to the extracts, highlighting their potential as alternative or adjunct antimicrobial agents. Variation in extraction yields and phytochemical profiles underscores the influence of geographic origin and extraction methodology.

Future directions include the isolation and detailed characterization of active compounds using GC–MS and LC-MS/MS, assessment of cytotoxicity, and exploration of potential synergistic effects with conventional antibiotics. Further research should also expand to a broader spectrum of pathogens, perform in vivo efficacy studies, evaluate safety and toxicity profiles, and investigate the underlying mechanisms of action to support the development of *M. chamomilla*-based antimicrobial therapies.

## Data Availability

All data generated and analyzed during this study are included in this published article.

## Abbreviations

* M. chamomilla*: *Matricaria chamomilla*
EOs: Essential oils
CFU: Colony forming unit
Azm15: Azithromycin
Gen: Gentamicin
Am: Amphotericin
CIP5: Ciprofloxacin
OF5: Ofloxacin
COT75: Clotrimazole
CFM5: Cefexime
LE5: Levofloxacin
NX10: Nalidixic acid
IPM10: Impepem
--: Not applicable
R: Resistant
MIC: Minimum inhibitory concentration
MBC: Minimum bactericidal concentration
* P. aeruginosa*: *Pseudomonas aeruginosa*
* S. aureus*: *Staphylococcus aureus*
